# Supplementary material for: Na Vacancy-Driven Phase Transformation and Fast Ion Conduction in W-Doped Na3SbS4 from Machine Learning Force Fields
Source: Chem Mater. 2024 Sep 19;36(19):9406–13. doi: 10.1021/acs.chemmater.4c00936 (PMC11467836; doi:10.1021/acs.chemmater.4c00936)
Supplement: Supplementary file 2 — cm4c00936_si_002.pdf [file cm4c00936_si_002.pdf]

SI for "Na Vacancy Driven Phase Transformation and Fast Ion Conduction in W-doped Na_3SbS_4 from Machine Learning Force Fields "

Johan Klarbring[1, 2, *] and Aron Walsh[1]

[1] *Department of Materials, Imperial College London, Exhibition Road, London SW7 2AZ, United Kingdom*
[2] *Department of Physics, Chemistry and Biology (IFM), Linköping University, SE-581 83, Linköping, Sweden*

(Dated: July 22, 2024)

I. VALIDATION OF THE ALLEGRO MLFF

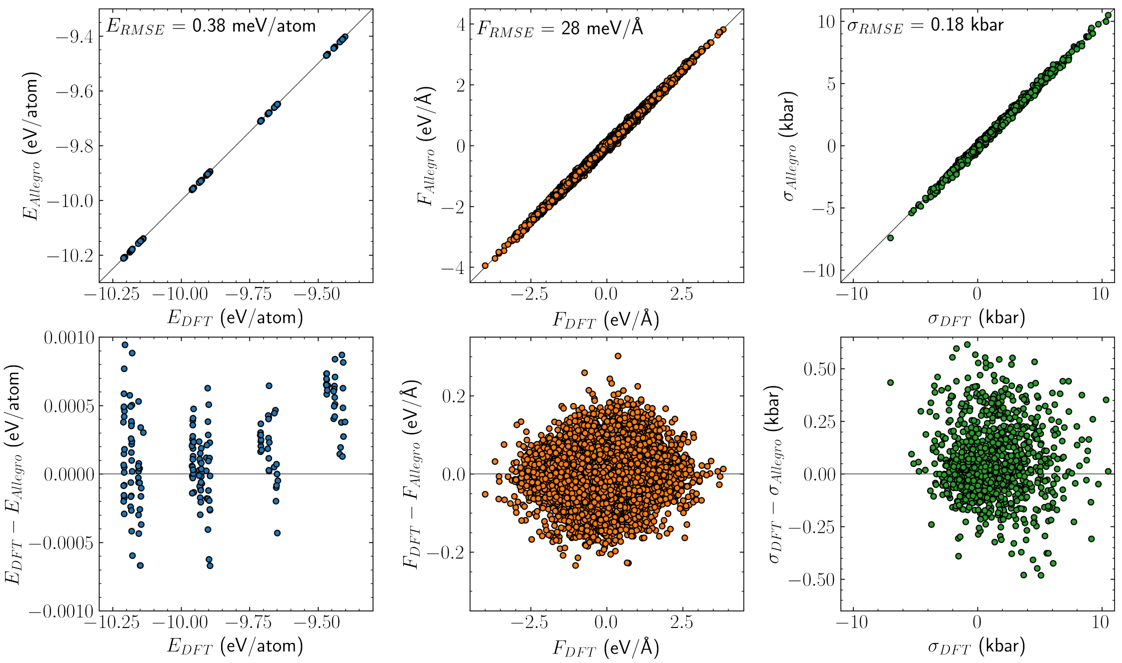

SI Fig. 1. (Top row) Parity plot for energies, force components and stress tensor components. (Bottom row) Errors distribution plots of energies, force components and stress tensor component. The 4 groupings in energy corresponds to systems with different number of W-dopants/Na-vacancies.

Na_3SbS_4				
Method	a_{cub} (Å)	a_{tetr}(Å)	c_{tetr}(Å)	c/a
r^2SCAN (static)	7.168	7.118	7.321	1.028
Allegro (static)	7.170	7.115	7.342	1.032
Allegro (300 K)	-	7.197	7.415	1.030
Exp (300 K)	-	7.160	7.290	1.018

TABLE I. Lattice constants for the cubic and tetragonal phases from DFT (r^2SCAN), Allegro and experiment[1]

* johan.klarbring@liu.se

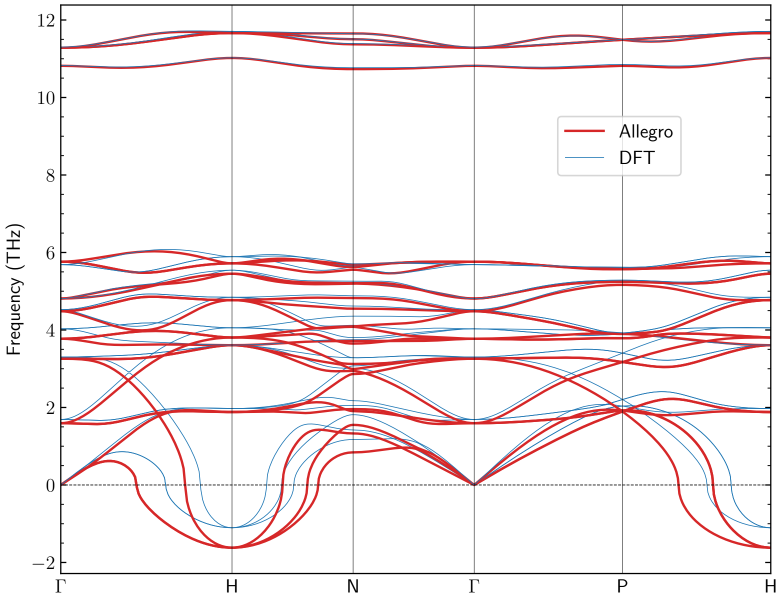

SI Fig. 2. Allegro and DFT phonon dispersion relation of cubic Na_3SbS_4.

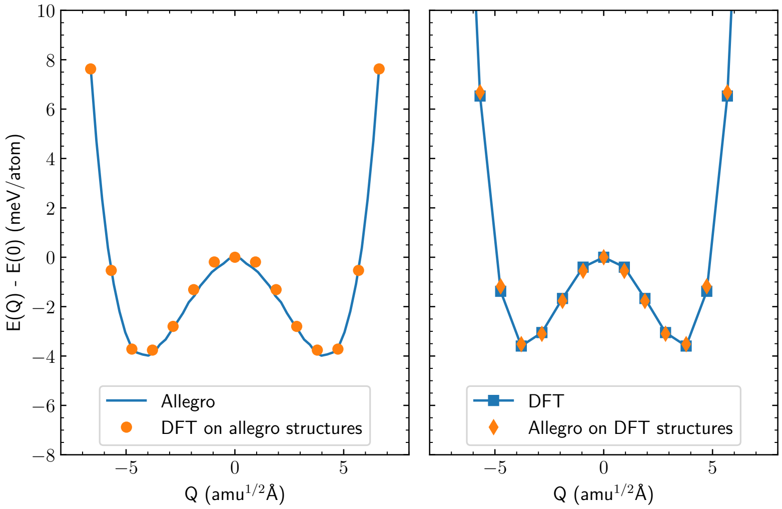

SI Fig. 3. Potential energy surface along the imaginary phonon mode at the H-point. Left plot follows the unstable phonon coordinate from the Allegro calculations, and compare to DFT energies at a few select points. Right plot follows the phonon coordinate from the DFT calculations and compares to the Allegro predicted energies. In each of the 4 cases, the energies are referenced to their respective values at Q=0.

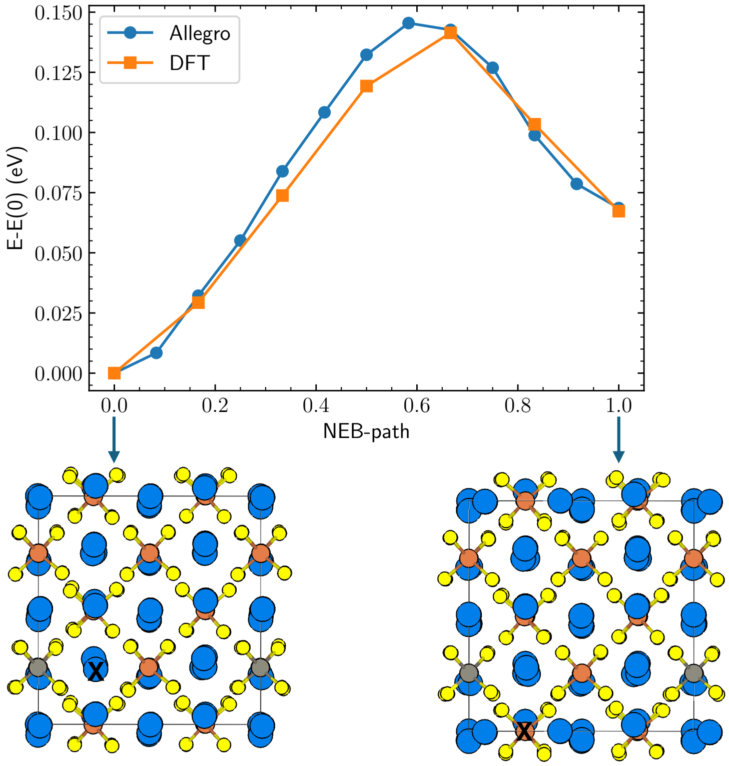

SI Fig. 4. Allegro and DFT Climbing image nudged elastic band (NEB) calculations of a Na-ion hop along the c direction in supercell with 1 W and 1 Na vacancy. The position of the vacancy in the initial and final Allegro relaxed structures is illustrated with an X. The calculations were performed using a 2×2×2 supercell with lattice parameters a=7.18 Å and c = 7.36 Å. Note that the energies in both curves are separately referenced to their respective values at the start of the path.

II. PHASE TRANSFORMATION

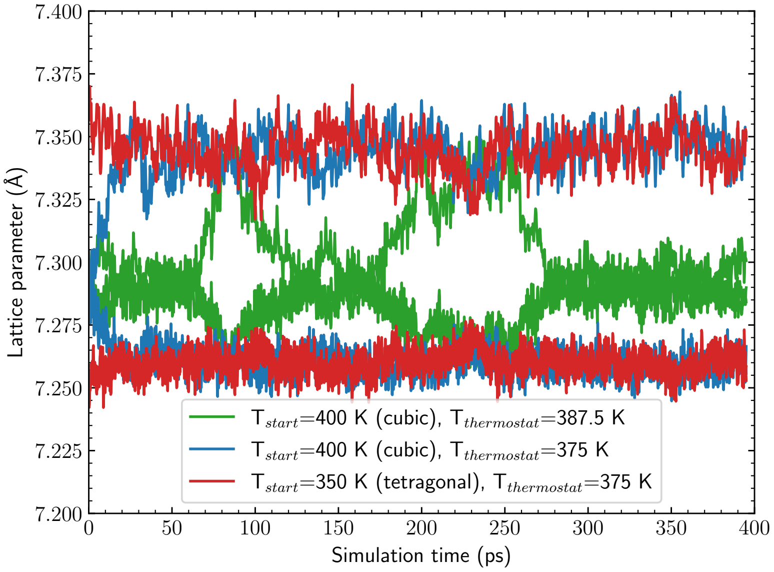

SI Fig. 5. Lattice parameters as a function of simulation time for a set of starting points and thermostat temperatures in $Na_{2.95}W_{0.05}Sb_{0.95}S_4$. The blue and red curves have the same thermostat temperature but different starting points, cubic and tetragonal, respectively. They can be seen to converge to the same steady state tetragonal lattice parameters and this temperature point can be safely identified as being in the tetragonal phase. For the green curve (400 K cubic starting point and thermostat set to 387.5 K), the system can be seen to jump between cubic and tetragonal configurations.

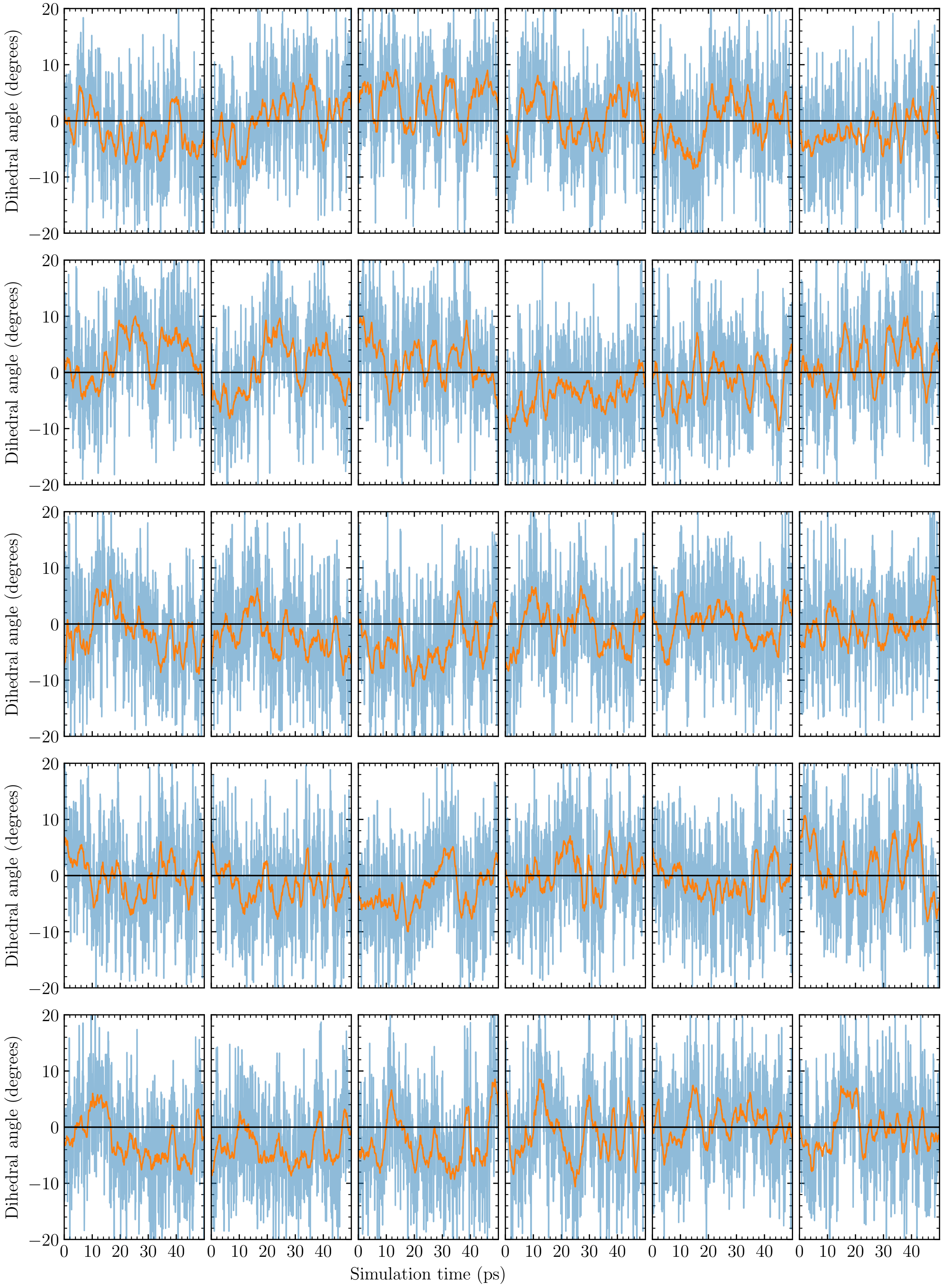

SI Fig. 6. Dihedral angles between sequential tetrahedra in 10 the % W substituted system at 400 K over 50 ps of simulation time.

III. DIFFUSION

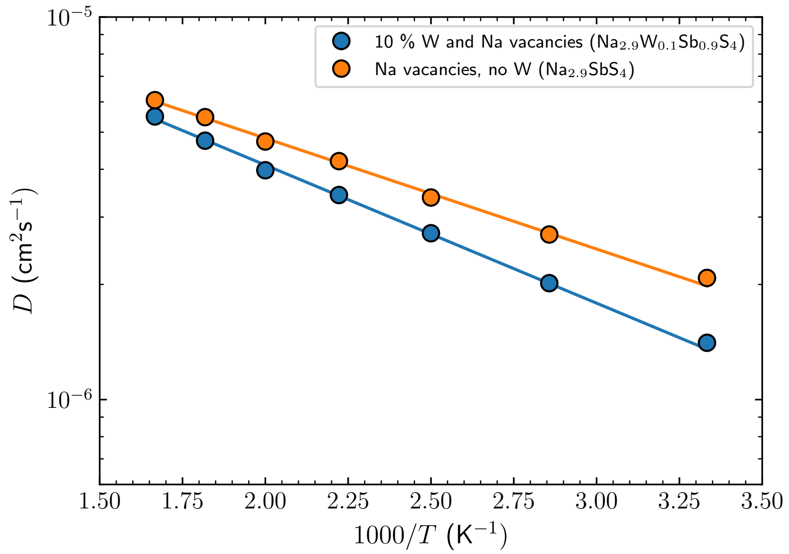

SI Fig. 7. Arrhenius plot of the self diffusion coefficient in the 10 % W-substituted system with compensating Na-vacancies ($Na_{2.9}W_{0.1}Sb_{0.9}S_4$ (blue markers) and in a Na-deficient system with the same number of Na-vacancies ($Na_{2.9}SbS_4$). Highlighting the weak detrimental effect that W-dopants has on the diffusion.

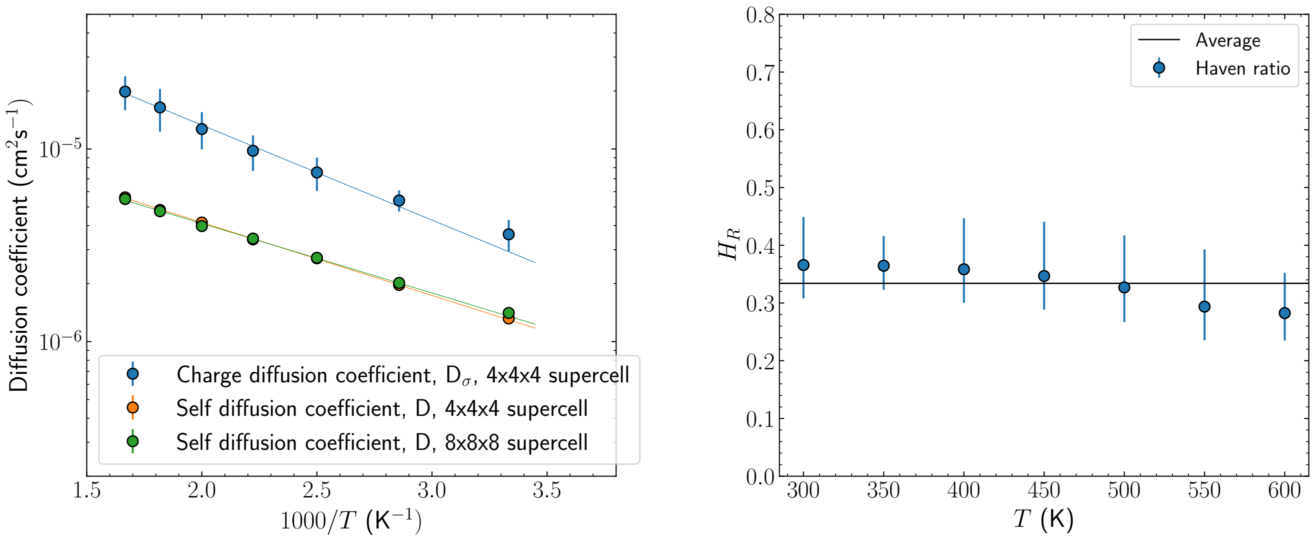

SI Fig. 8. (left plot) Arrhenius plots of the self diffusion coefficients calculated in 4×4×4 and 8×8×8 supercells and charge diffusion coefficient calculated in an 4×4×4 supercell. (Right plot) Temperature dependence of the Haven ratio, H_R. The black line shows the average value of H_R over temperature.

[1] O. Maus, M. T. Agne, T. Fuchs, P. S. Till, B. Wankmiller, J. M. Gerdes, R. Sharma, M. Heere, N. Jalarvo, O. Yaffe, M. R. Hansen, and W. G. Zeier, Journal of the American Chemical Society **145**, 7147–7158 (2023).
